# Accuracy and Reproducibility of Laboratory Diffuse Reflectance Measurements with Portable VNIR and MIR Spectrometers for Predictive Soil Organic Carbon Modeling

**DOI:** 10.3390/s22072749

**Published:** 2022-04-02

**Authors:** Sebastian Semella, Christopher Hutengs, Michael Seidel, Mathias Ulrich, Birgit Schneider, Malte Ortner, Sören Thiele-Bruhn, Bernard Ludwig, Michael Vohland

**Affiliations:** 1 Geoinformatics and Remote Sensing, Institute for Geography, Leipzig University, 04103 Leipzig, Germany; sebastian.semella@gmail.com (S.S.); michael.seidel@uni-leipzig.de (M.S.); mathias.ulrich@uba.de (M.U.); 2 Remote Sensing Centre for Earth System Research, Leipzig University, 04103 Leipzig, Germany; 3 German Centre for Integrative Biodiversity Research (iDiv) Halle-Jena-Leipzig, 04103 Leipzig, Germany; 4 Physical Geography, Institute for Geography, Leipzig University, 04103 Leipzig, Germany; bschneid@rz.uni-leipzig.de; 5 Soil Science, Faculty of Spatial and Environmental Sciences, University of Trier, 54286 Trier, Germany; ortner@uni-trier.de (M.O.); thiele@uni-trier.de (S.T.-B.); 6 Department of Environmental Chemistry, University of Kassel, 37213 Witzenhausen, Germany; bludwig@uni-kassel.de

**Keywords:** visible-to-near infrared, mid-infrared, portable, spectroscopy, soil organic carbon, dry combustion, uncertainty, ring trial, partial least-squares regression, Monte Carlo cross-validation

## Abstract

Soil spectroscopy in the visible-to-near infrared (VNIR) and mid-infrared (MIR) is a cost-effective method to determine the soil organic carbon content (SOC) based on predictive spectral models calibrated to analytical-determined SOC reference data. The degree to which uncertainty in reference data and spectral measurements contributes to the estimated accuracy of VNIR and MIR predictions, however, is rarely addressed and remains unclear, in particular for current handheld MIR spectrometers. We thus evaluated the reproducibility of both the spectral reflectance measurements with portable VNIR and MIR spectrometers and the analytical dry combustion SOC reference method, with the aim to assess how varying spectral inputs and reference values impact the calibration and validation of predictive VNIR and MIR models. Soil reflectance spectra and SOC were measured in triplicate, the latter by different laboratories, for a set of 75 finely ground soil samples covering a wide range of parent materials and SOC contents. Predictive partial least-squares regression (PLSR) models were evaluated in a repeated, nested cross-validation approach with systematically varied spectral inputs and reference data, respectively. We found that SOC predictions from both VNIR and MIR spectra were equally highly reproducible on average and similar to the dry combustion method, but MIR spectra were more robust to calibration sample variation. The contributions of spectral variation (ΔRMSE < 0.4 g·kg^−1^) and reference SOC uncertainty (ΔRMSE < 0.3 g·kg^−1^) to spectral modeling errors were small compared to the difference between the VNIR and MIR spectral ranges (ΔRMSE ~1.4 g·kg^−1^ in favor of MIR). For reference SOC, uncertainty was limited to the case of biased reference data appearing in either the calibration or validation. Given better predictive accuracy, comparable spectral reproducibility and greater robustness against calibration sample selection, the portable MIR spectrometer was considered overall superior to the VNIR instrument for SOC analysis. Our results further indicate that random errors in SOC reference values are effectively compensated for during model calibration, while biased SOC calibration data propagates errors into model predictions. Reference data uncertainty is thus more likely to negatively impact the estimated validation accuracy in soil spectroscopy studies where archived data, e.g., from soil spectral libraries, are used for model building, but it should be negligible otherwise.

## 1. Introduction

Over the last two decades, visible-to-near infrared (VNIR) and mid-infrared (MIR) soil spectroscopy have been established as rapid, inexpensive and eco-friendly methods of soil analysis [1], in particular for the determination of soil organic carbon (SOC). Recently introduced lightweight, portable MIR spectrometers [2,3,4] have further broadened the scope of infrared soil analysis by potentially allowing on-site applications of MIR soil analysis, complementing long-established portable VNIR instruments [5,6] and offering additional flexibility in laboratory settings.

Determining soil properties from soil reflectance data requires the development of multivariate calibrations, i.e., regression models that relate the measured spectral signal to the soil property of interest using reference values obtained from primary analytical methods. VNIR and MIR estimates of soil properties are generally less accurate than the corresponding laboratory-analytical data retrieved with standardized measuring protocols [7]. For many soil properties such as SOC, soil nitrogen (N), clay and carbonate contents, however, spectroscopic models can be sufficiently accurate to partially replace standard laboratory methods in routine soil surveying [8]. The common assumption that spectral predictions cannot be better than the reference method used for the calibration is also not strictly true since multivariate modeling with many predictor variables is theoretically capable of compensating for noise in laboratory reference data [9,10].

An issue arising in this context is that error metrics for spectroscopic models are computed against the analytical reference data under the assumption that these data are essentially error-free. This may lead to potentially misleading error estimates as it is the deviation from the analytical reference data that is actually measured, which includes the uncertainty inherent in the analytical data itself. At the same time, it would be incorrect, however, to blame significant errors or poor model performance on the usually unknown laboratory error without experimental proof [11]. This documents the need to assess the uncertainty inherent in laboratory reference data as one error source in spectroscopic modeling and thus to distinguish between true and apparent prediction errors, with the latter arising, for example, from inaccurately measured reference values [9,10].

In addition to errors in the reference data, uncertainty in the soil property estimates of spectroscopic models arises from deficits in the accuracy and reproducibility of the respective spectral measurements, determined, e.g., by instrumental characteristics, sample type and preparation, and also from error sources of the model building procedure [12]. For bench-top VNIR and MIR spectrometers, the reproducibility of spectral measurements can be considered high on homogenized sampling material due to standardized measurement protocols as well as fixed illumination and sampling setups. For portable VNIR instruments, Kuester et al. [13] showed high repeatability for surface reflectance measurements under well-controlled environmental conditions. In the case of portable MIR spectrometers, previous studies have also shown that these instruments can measure soil spectra with a similar quality to bench-top MIR spectrometers and allow calibrations of soil properties with comparable accuracy [3,4,6,14,15]. For instance, Hutengs et al. [4] showed that MIR calibrations developed with spectra measured by different operators using an Agilent 4300 Handheld FTIR yielded very similar root-mean-square errors (RMSE) for SOC (1.9–2.0 g·kg^−1^), N (0.17–0.21 g·kg^−1^) and the clay content (1.77–2.03%). Good reproducibility of handheld MIR measurements was also reported by Janik et al. [6] on finely-ground soil material, as indicated by a spectral repeatability function calculated from linear correlation between pairs of spectra.

As a consequence of the described sources of uncertainty, a complete evaluation of spectroscopic modeling results requires a separate error assessment of the analytical reference measurements, in addition to an assessment of the spectral repeatability. This may be achieved through the replication of reference and spectral measurements on the same samples, as demonstrated, e.g., by Aastveit et al. [16], who used this approach to compare the variations of reference measurements and corresponding spectroscopic estimates to judge the robustness of spectral estimation in forage breeding.

In soil spectroscopy, Stevens et al. [17] re-analyzed a set of 25 soil samples to calculate the reproducibility error of reference measurements during the development of the European LUCAS soil database. For SOC determined by the dry combustion laboratory method, they reported a reproducibility error of ~2.0 g·kg^−1^ against an error of ~1.8 g·kg^−1^ for VNIR-based estimates, from which they concluded that only a small error term could be attributed to spectral measurement conditions. Replication is also useful to improve the validation sample accuracy as averaging unbiased repeated measurements made with the same laboratory method will tend to reduce the random component of the error [11]. Given the additional workload, this is rarely done in practice. Alternatively, statistical corrections of reference measurements may be feasible but require further assumptions about the characteristics of the error term [18]. More recently, Ellinger et al. [12] analyzed the impact of spectral variation and repeated SOC measurements on the validation of VNIR spectroscopic models and found that an error component of ΔRMSE ~0.4 g·kg^−1^ could be attributed to the combined uncertainty introduced by variation in spectral measurements and reference SOC values compared to spectra and SOC data both averaged.

The widespread application of soil spectroscopy has recently received new impetus through the establishment of large spectral libraries and the introduction of portable MIR instruments. Addressing the various sources of uncertainty and their implications in spectral modeling is thus becoming increasingly important, e.g., to contribute to the development of standardized protocols for soil spectroscopic measurements and calibration development [12].

In the present study, we analyzed the impact of uncertainty in spectral measurements with portable spectrometers and analytical reference data on SOC calibrations in both the VNIR and MIR spectral ranges. To this end, we collected VNIR, MIR and SOC data in triplicate for topsoil samples collected from 75 agricultural fields and developed VNIR and MIR models to estimate SOC for various combinations of spectral measurement series and SOC laboratory measurements, with the aim to (1) determine the impact of uncertainty in the reference SOC measurements on VNIR and MIR calibrations, (2) evaluate whether spectral variation across repeated measurements or uncertainty in the reference soil property data contributes more to overall modeling accuracy and (3) assess which spectral range is more robust regarding the reproducibility of spectral measurements and the overall stability of SOC calibrations.

## 2. Materials and Methods

### 2.1. Soil Sampling and Pre-Treatment

**Figure 1:**
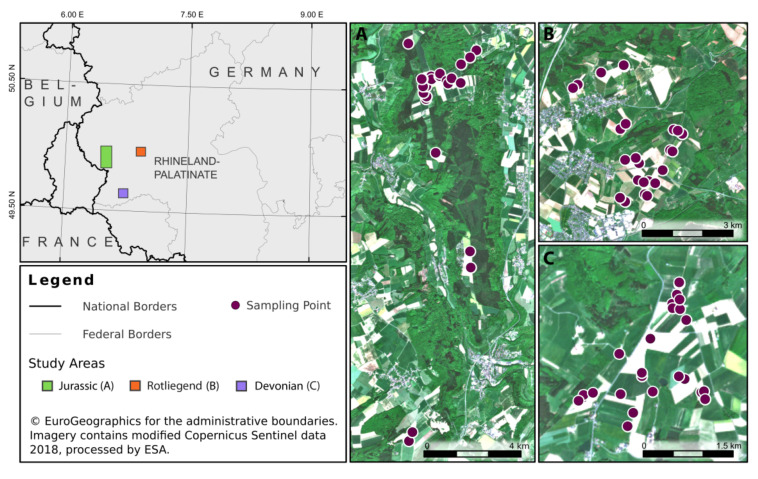
Soil sampling sites in western Rhineland-Palatinate, Germany, stratified for different geological origins, with individual sampling locations (*n* = 75) illustrated in panels A–C.

Soil samples were collected from the top layer (A_p_ horizon, 0–25 cm) of 75 agricultural fields in western Rhineland-Palatinate, Germany (Figure 1). The sampling region is characterized by a diverse set of soil parent materials derived from (i) Devonian clay schists, (ii) Permian siltstones and fine sandstones of the Rotliegend group and (iii) Jurassic sandstones of the Luxembourg formation [19,20]. We took 25 soil samples from each of these parent material strata to cover a broad range of soil texture classes, with sand and clay contents ranging from 20.1 to 89.3% and 5.7 to 34.6%, respectively, and SOC values ranging from 6.18 to 35.54 g·kg^−1^. Sampled soils were free of carbonates [21]; to prevent sample (pseudo-)replication, all soils were taken from different agricultural fields.

The collected soil material was air-dried, sieved (<2 mm) and thoroughly mixed. Then, 15 g of each sieved soil sample was ground using a planetary mill (PM 200, RETSCH GmbH, Haan, Germany) for five minutes. Analytical SOC measurements (Section 2.2), along with VNIR and MIR data acquisition (Section 2.3), were carried out on subsamples of the finely ground material (<10 µm) to minimize the impacts of material heterogeneity on the uncertainty analysis. To this end, each sample was split into nine subsamples to enable three independent repetitions for each method.

### 2.2. Laboratory-Analytical Determination of SOC

SOC values of those subsamples that had been put aside for reference measurements were determined independently by three laboratories, using the Vario EL Cube (Elementar, Langenselbold, Germany) or the Vario Max CHN (Elementar, Langenselbold, Germany) elemental analyzers by means of the dry-combustion standard method [22]. In brief, all carbon-containing compounds of the portioned soil material were converted into carbon dioxide by high-temperature oxidation (depending on the instrument, at 900 or 950 °C) in a pure-oxygen atmosphere. The final quantification of carbon dioxide was then realized with a thermal conductivity detector. A more detailed description of the principles of the dry-combustion measurement process can be found, for example, in Chatterjee et al. [23].

The total carbon content (TOC; in g C per kg of soil on an oven-dry basis) can be considered equivalent to the SOC for soils without contributions of carbonate-C. SOC reference values provided by the different measurement series from the three laboratories are hereafter denoted with Lab_1_, Lab_2_ and Lab_3_.

### 2.3. VNIR and MIR Soil Reflectance Measurements

VNIR data were measured with an ASD FieldSpec 4 (Malvern Panalytical Ltd, Malvern, UK) and MIR spectra with an Agilent 4300 Handheld FTIR (Agilent Technologies, Santa Clara, CA, USA) spectrometer (Table 1). Corresponding to dry-combustion reference analyses, we measured three sets of independent VNIR and MIR spectral data (different operators, randomized sample order), hereafter denoted with the numbers 1–3.

For each of the three sets, measurements with the ASD FieldSpec 4 (350–2500 nm) were carried out with the instrument sensor mounted on a tripod (nadir view, 25° field-of-view, ~15 cm distance from the sample) and the soil sample spread out in a petri dish. We used a 100-W light source (Spectral Evolution, Haverhill, MA, USA) to illuminate the sample (45° illumination zenith angle) and calibrated the spectroradiometer with a Zenith Polymer^®^ reference panel at five-minute intervals. In each set, all samples were scanned twice (with different subsample material in the petri dish) with 75 internal scans each. Finally, these two individual spectra were averaged into one single spectrum.

The Agilent 4300 Handheld FTIR (4000–650 cm^−1^) spectrometer was used with a diffuse reflectance interface and a spot diameter of 2 mm. For measurements, we filled a small sample cup with soil material; stable viewing angles (~0° zenith angle) and distances (<0.5 mm) between the sample material and sampling interface were achieved by means of a small sample holder (see [4] for a more detailed description of the measuring setup). Measurements were again repeated twice with a different subsample material. We recorded MIR spectra at a 4 cm^−1^ spectral resolution with 64 co-added scans (Table 1); the instrument was calibrated with a manufacturer-provided gold-plated reference cap at five-minute intervals. Individual MIR spectra were averaged into a single spectrum for each sample.

VNIR and MIR reflectance spectra (R) were finally converted into absorbance units (A) by A = −log_10_(R). We excluded the 350–400 nm (VNIR) and 800–650 cm^−1^ (MIR) spectral regions from further analysis due to low signal-to-noise levels. Spectra were resampled to 1 nm and 2 cm^−1^ increments, which resulted in 2100 data points for VNIR and 1600 data points for MIR spectra.

### 2.4. Evaluating Uncertainties in Analytical and Spectroscopic Measurements

Analytical laboratory SOC measurements from the three laboratories were compared by calculating the root-mean-square error (RMSE), mean error (bias) and coefficient of determination (R^2^) between pairs of individual series (Lab_1_–Lab_3_) and between these series and their averaged values (Lab_AVG_), to evaluate the reproducibility and uncertainty associated with the SOC reference data. To compare the reproducibility of the spectral recordings, we used the spectral repeatability function *S_r_*, which was proposed by Janik et al. [6] as a modification of Pearson’s coefficient of correlation *r* (where *r* is calculated between scans of replicate samples), calculated as $S_{r}=1/\left(1-r\right)$, since in cases where only relatively small variations between spectra occur, *r* is relatively insensitive [6]. Increasing values for *S_r_* indicate high similarities between pairs of spectra.

### 2.5. Examining the Influence of Uncertainties on SOC Modeling Results

**Figure 2:**
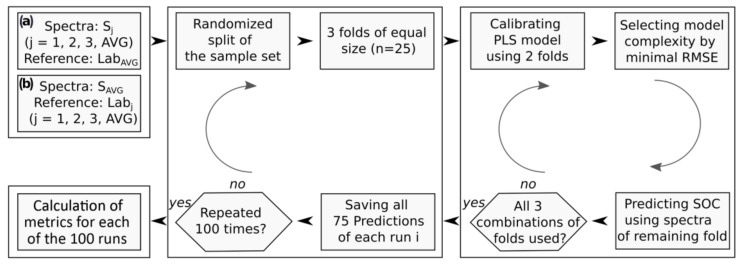
Processing scheme of the repeated cross-validation (CV) approach to study uncertainties from (**a**) spectral measurements and (**b**) reference SOC data.

VNIR- and MIR-based models for SOC estimation were calibrated by means of partial least-squares regression (PLSR) and evaluated in a Monte Carlo cross-validation (CV) approach with k = 100 repetitions. To this end, the spectral dataset with *n* = 75 samples was partitioned into m = 3 folds with 25 samples each, selected through stratified random sampling with respect to the soil parent material (Figure 2). PLSR models were calibrated on two of these folds with the number of latent variables determined in an (internal) leave-one-out CV (minimum RMSE criterion). The calibrated model was then run on the spectra in the remaining fold to predict SOC contents. This procedure was repeated (with other calibration folds) so that all samples were in the validation set once (i.e., we pooled *n* = 75 validation samples). We repeated this CV approach 100 times so that we retrieved averaged estimates for each sample and standard deviations from these 100 runs. PLSR models were fitted on square root-transformed SOC values to center the data distribution [24]. Individual model predictions were back-transformed to the original scale before calculating model performance metrics.

To assess the contributions of different sources of uncertainty, i.e., variation in VNIR and MIR measurements and laboratory SOC reference data versus the estimated validation error in spectroscopic modeling, we carried out the above-described analysis with different combinations of spectral data and SOC reference measurements. First, we used spectra averaged from VNIR_1_–VNIR_3_ (VNIR_AVG_) and from MIR_1_–MIR_3_ (MIR_AVG_) together with averaged reference values (Lab_AVG_), which represents the best-case scenario for spectral modeling with the theoretically most accurate spectral and reference data. Then, we repeated the calibrations with Lab_AVG_ reference data, but with each spectral data series (VNIR_1_–VNIR_3_, MIR_1_–MIR_3_) individually, to assess the influence of uncertainty associated with variation across repeated spectral measurements. Finally, we carried out the calibrations with averaged spectral data (VNIR_AVG_ and MIR_AVG_), varying the SOC reference data (Lab_1_–Lab_3_) instead. All computations were carried out in the R statistical software (R 4.0.3; [25] using the packages “pls” (v2.8; [26]) and “prospectr” (v0.2.1; [27])).

## 3. Results

### 3.1. Comparison of Analytical SOC Measurements by Dry Combustion

Analytical SOC measurements from the three laboratories are summarized in Table 2. The organic carbon contents in the sampled soils ranged on average (Lab_AVG_) from 6.2 to 35.5 g·kg^−1^ with a mean of 14.6 g·kg^−1^ and a standard deviation of 7.9 g·kg^−1^. The distribution of SOC values was slightly right-skewed overall, with a skewness of 0.53. Individual laboratory measurement (Lab_1_–Lab_3_) series agreed very well with respect to the overall SOC distribution. Regarding the geologic stratification of the sampled soils, the lowest average SOC contents were observed in the Rotliegend (10.2 g·kg^−1^), with the highest in the Devonian (26.2 g·kg^−1^) and the Jurassic falling in-between (14.7 g·kg^−1^).

The sample-wise agreement of the three laboratory measurement series was also excellent (Table 3), with R^2^ values between any two series greater than 0.99. The RMSE between SOC values determined by the three laboratories was low (0.36–0.80 g·kg^−1^); Lab_1_ and Lab_2_ series agreed particularly well (RMSE = 0.36 g·kg^−1^, bias = 0.10 g·kg^−1^). The deviation of Lab_3_ from Lab_1_ and Lab_2_ was larger (RMSE = 0.78 and 0.80 g·kg^−1^, respectively), with a major part of the error attributable to a bias of −0.59 and −0.69 g·kg^−1^, i.e., the Lab_3_ SOC values were systematically larger. The bias was still relatively small overall but statistically significant (paired *t*-test, *p* < 0.001).

At the level of individual samples, the absolute largest deviation between any two laboratory values was 2.57 g·kg^−1^. Variation between laboratory measurements at the individual sample level had a tendency to increase from low to high SOC values, i.e., the sample-wise standard deviation of the three replicate measurements increased with SOC content from, on average, SD = 0.21 g·kg^−1^ for SOC contents <10 g·kg^−1^, to SD = 0.65 g·kg^−1^ for SOC contents >25 g·kg^−1^.

### 3.2. Evaluation of VNIR and MIR Reflectance Measurements

**Figure 3:**
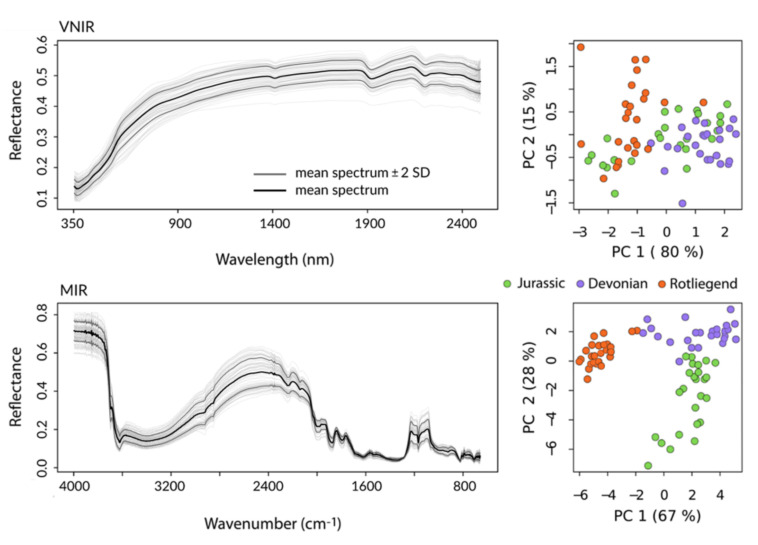
VNIR and MIR spectra of the collected soil samples (*n* = 75) in light gray showing the total spectral variation across the sampled soil data; the mean spectrum (black) and its associated ±2 standard deviation (SD) envelope (dark gray) illustrate prominent absorption features in the spectra. Right-hand panel shows the corresponding location of the spectra in the principal component feature space, color-coded by underlying geologic strata.

The recorded VNIR and MIR spectra (Figure 3) were characterized by high spectral diversity corresponding to the heterogeneous composition of the collected soil sample set. Compared to the VNIR data, MIR spectra depicted significantly more well-defined absorption bands, e.g., features directly and indirectly linked to SOC (2950–2870 cm^−1^), the clay content (clay minerals; 3600–3700 cm^−1^) and sand content (silicates, predominantly quartz; 2000–1790 cm^−1^, 1280–1070 cm^−1^).

Accordingly, the MIR data contained considerably more information about the mineral and organic composition of the sampled soils, which allowed clear separation of the samples along geologic strata through principal component analysis (PCA; Figure 3). In the PCA feature space of the MIR data, individual samples formed well-defined compact clusters. In contrast, soil samples showed extensive overlap in the VNIR for geologically defined groups; Devonian and Rotliegend soils could be separated well but were intersected by the point cloud of the Jurassic samples.

The spectral variability between VNIR and MIR replicate measurements with the portable spectrometers was evaluated using the spectral variability function *S_r_*, calculated between pairs of corresponding spectra for the different combinations of the measurement series 1–3 (Table 4). Absolute values of *Sr* for VNIR data were significantly larger than for MIR data on average, with values of *S_r_* from 27,615 to 32,351 (VNIR) and from 2211 to 2729 (MIR), respectively.

However, a direct comparison of these values would be misleading since *S_r_* is a function of the correlation coefficient between two spectra, which inevitably tends to be larger for the VNIR than for the MIR data due to the presence of considerably greater autocorrelation in the former. Using the coefficient of variation (CV_%_) for comparison between the series instead, which normalizes the standard deviation of the *S_r_* distribution by its mean, indicated comparable reproducibility of VNIR and MIR spectral measurements, with a moderate advantage for the MIR instrument in two of the three pairwise comparisons, as CV_%VNIR_ ranged from 61.2 to 68.7% and CV_%MIR_ was between 48.4 and 65.0%. Variation of *S_r_* within the measured series, i.e., among spectral duplicates, was also comparable between the VNIR and MIR instruments (CV_%VNIR_ = 49.5–65.5, CV_%MIR_ = 50.7–69.0).

### 3.3. Accuracy of Predictive VNIR and MIR Models

**Figure 4:**
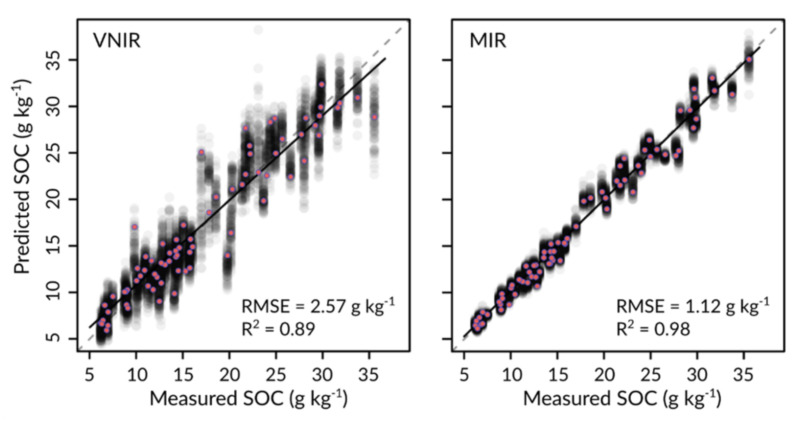
Pooled validation results of VNIR and MIR SOC estimates for the ‘best-case’ scenario with averaged spectral data from all measurement series (VNIR_AVG_ and MIR_AVG_) and averaged SOC reference data from all three laboratories. Red points correspond to the runs with average RMSE values to illustrate typical errors of VNIR- or MIR-predicted SOC values. Transparent gray points represent predictions from all 100 cross-validation runs, indicating the predictive uncertainty associated with each data point.

Both VNIR and MIR spectra allowed accurate predictions of SOC content for the sampled soils, although the MIR results were significantly better in direct comparison (Table 5). For the ‘best-case’ scenario with minimal spectral (VNIR_AVG_, MIR_AVG_) and SOC reference data (Lab_AVG_) variability, SOC prediction errors averaged 2.57 g·kg^−1^ for VNIR and 1.12 g·kg^−1^ for MIR data. Compared with the mean RMSE between any two respective laboratory-replicate measurements (~0.65 g·kg^−1^), the MIR results were thus less accurate by a factor of ~2, and the VNIR results by a factor of ~4. The bias of the predictive models was small in both spectral ranges, but the spectral models calibrated with MIR spectra were more robust than those based on VNIR spectra, indicated by a markedly smaller RMSE standard deviation of SD_RMSE,MIR_ = 0.08 g·kg^−1^ for MIR models against SD_RMSE,VNIR_ = 0.25 g·kg^−1^ for VNIR models. Accordingly, the VNIR estimates for individual samples were not only less accurate on average but also scattered significantly more across all cross-validation runs (Figure 4).

### 3.4. Impacts of Spectral Variability and SOC Reference Data on Validation Accuracy

The influence of spectral variability and reproducibility of VNIR and MIR measurements on SOC predictions was analyzed by systematically varying the spectral measurement series used for model calibrations and predictions (validation), using the averaged SOC from all three laboratories as calibration and validation reference data (Table 6). Compared to the ‘best-case’ scenario with averaged spectral data from three repeated measurement series described in the previous section, SOC predictions were less accurate for any given combination of individual spectral datasets.

For the respective VNIR models, average RMSEs ranged from 2.65–2.91 g·kg^−1^ with a mean of 2.80 g·kg^−1^ and an error ~9% larger (ΔRMSE = 0.23 g·kg^−1^) than for models using VNIR_AVG_ in both calibration and validation (2.57 g·kg^−1^). RMSEs for combinations of individual MIR datasets were between 1.30 and 1.48 g·kg^−1^ with a mean of 1.40 g·kg^−1^, corresponding to an increased error of ~25% (ΔRMSE = 0.28 g·kg^−1^) compared to model calibration and validation with MIR_AVG_ (1.12 g·kg^−1^).

The variation in absolute RMSEs, however, was equally low for both instruments with ΔRMSE_VNIR_ <0.26 g·kg^−1^ (CV_%_ ~3.3%) and ΔRMSE_MIR_ <0.18 g·kg^−1^ (CV_%_ ~4.4%). Using the same individual spectral datasets for calibration and validation did not lead to better validation accuracies for either spectral range (RMSE_VNIR_ = 2.81 g·kg^−1^, RMSE_MIR_ = 1.43 g·kg^−1^) compared to mixed calibration-validation data. RMSE standard deviations for all dataset combinations within a given spectral range were also very similar (SD_RMSE,VNIR_ = 0.22–0.26 g·kg^−1^, SD_RMSE,MIR_ = 0.07–0.11 g·kg^−1^) and in line with models using VNIR_AVG_ and MIR_AVG_ spectra (SD_RMSE,VNIR_ = 0.25 g·kg^−1^, SD_RMSE,MIR_ = 0.08 g·kg^−1^).

We finally analyzed the impact of the laboratory SOC data on the VNIR and MIR model validation accuracy (Table 7) by varying the SOC reference data in model calibration and validation, keeping the spectral inputs constant (VNIR_AVG_ and MIR_AVG_). Validation RMSEs in this experiment ranged from 2.56–2.73 g·kg^−1^ for VNIR models and 1.12–1.41 g·kg^−1^ for MIR models, respectively, corresponding to an error increase of ~3% and ~12% on average compared to the ‘best-case’ models. Increases in validation RMSE, however, were limited to calibration-validation SOC data combinations involving Lab_3_ data, which were biased upward relative to Lab_1_ and Lab_2_, with ΔRMSE values of 0.11–0.16 g·kg^−1^ (VNIR) and 0.25–0.29 g·kg^−1^ (MIR), which was lower than the uncertainty associated with varying spectral inputs for the VNIR models and comparable in magnitude to MIR models. Varying the unbiased Lab_1_ and Lab_2_ SOC reference values during model calibration and validation, on the other hand, did not decrease validation RMSEs in either spectral range. The spread of the RMSE distributions was also largely unaffected for any combination of SOC reference data (SD_RMSE,VNIR_ = 0.24–0.27 g·kg^−1^, SD_RMSE,MIR_ = 0.08–0.10 g·kg^−1^), similar to the results from models with varying spectral inputs.

## 4. Discussion

Our inter-laboratory comparison of analytical SOC measurements confirmed the high accuracy and reproducibility of the dry-combustion reference method. SOC measurement uncertainty in our study, i.e., the variation between the three laboratories (0.36–0.80 g·kg^−1^) and their variation from the overall mean (0.30–0.52 g·kg^−1^), was generally lower than anticipated based on other inter-laboratory comparisons [17,28,29]. Proficiency-testing data compiled by the Association of German Agricultural Investigation and Research Institutes (VDLUFA, [28]), for example, gives expected measurement uncertainties of 0.62–2.19 g·kg^−1^ for SOC contents between 6 g·kg^−1^ and 36 g·kg^−1^. Similarly, Stevens et al. [17] reported uncertainty of 2.0 g·kg^−1^ for repeated laboratory analyses of mineral soils, with an average SOC content of 29.4 g·kg^−1^ for data collected for the LUCAS soil library, while an interlaboratory comparison of forest soil samples (Bs horizon, 35.16 g·kg^−1^ average SOC) by Ross et al. [29] yielded a SOC measurement uncertainty of 2.31 g·kg^−1^. The lower inter-laboratory uncertainty in our study presumably resulted from carrying out the soil pre-treatment before sending the samples to the different laboratories for dry-combustion analysis, as homogenizing the soil samples through fine-grinding likely removed residual sampling variation that would be present in sub-sampled fresh or sieved soils.

Spectral measurements with the handheld VNIR and MIR instruments and the derived predictive SOC models were also highly reproducible, as indicated by both the spectral repeatability analysis and the low variation in validation RMSEs when interchanging individual spectral measurement series for model calibration and prediction. The absolute variation of RMSEs was equally low for both VNIR (ΔRMSE_VNIR_ < 0.26 g·kg^−1^, CV_%_ ~3.3%) and MIR (ΔRMSE_MIR_ < 0.18 g·kg^−1^, CV_%_ ~4.4%) data, suggesting comparable reproducibility of repeat spectral measurements and SOC calibrations on finely ground soil material.

The latter qualification is relevant given the importance of sample pre-treatment for the accuracy and reproducibility of VNIR and MIR measurements and derived SOC models [30,31,32,33,34,35,36]. Fine-grinding generally increases predictive accuracies and is especially relevant for MIR spectroscopy, where the amount of scanned soil material is lower and thus likely less representative without careful homogenization due to smaller beam apertures and shallower penetration depths for MIR instrumentation [2]. Janik et al. [6], for example, reported progressively improving spectral reproducibility for MIR spectra, scanned with the handheld ExoScan (Agilent 4100) instrument (6000–650 cm^−1^), with increasing sample homogenization, ranging from *S_r_* = 67 for intact soils to *S_r_* = 144 and *S_r_* = 293 for sieved (<2 mm) and fine-ground (~0.1 mm) samples, respectively. The corresponding *S_r_* values we calculated for our MIR datasets (average *S_r_* = 1428; 4000–800 cm^−1^) were markedly higher, in accordance with the more finely ground samples (~10 µm), although the covered spectral ranges were not identical, complicating a direct comparison. In this context, we recently also found that MIR SOC models were significantly impacted by sample pre-treatment differences (finely ground vs. sieved), whereas VNIR models were hardly affected [5]. Accordingly, our finding that VNIR and MIR handheld instruments provide measurements with comparable spectral reproducibility and accuracy is probably limited to applications with finely ground sample material.

Moreover, the repeated cross-validation analysis showed that model calibrations with MIR spectra were more robust against calibration sample selection than VNIR spectra, as measured by the RMSE standard deviation across cross-validation runs. This can be linked to the presence of more specific and well-defined absorption features in the MIR domain [37,38,39,40], allowing more stable PLSR model calibrations that rely on fewer latent variables [5,40]. Despite the high reproducibility of individual VNIR and MIR spectral measurements and their corresponding SOC calibrations, averaging all spectral datasets recorded on different soil sub-samples still allowed some improvement of predictive accuracies; in line with previous studies [4,12], our data were comparable in absolute values in both spectral ranges (ΔRMSE_VNIR_ = 0.23 g·kg^−1^, ΔRMSE_MIR_ = 0.28 g·kg^−1^). As soil samples can be scanned reasonably quickly with both spectrometers (~30 s or less per scan), collecting spectra on a larger number of soil subsamples may be advisable. However, the additional workload would have to be weighed against the relatively minor improvements, at least for spectra collected under laboratory conditions on finely ground material. A larger number of spectral replicates should definitely be considered, however, for in-situ applications of portable spectrometers on much more heterogeneous samples [5,41,42].

The effect of varying the calibration and validation of SOC reference data on model accuracy was also small and effectively only present when Lab_3_ SOC values, which were biased slightly upward, were used in combination with Lab_1_ and Lab_2_ data, respectively. In this case, validation RMSEs increased by ~0.14 g·kg^−1^ (ΔRMSE_VNIR_) and ~0.26 g·kg^−1^ (ΔRMSE_MIR_). It thus appears that the random error component of the SOC reference data is effectively compensated for during model calibration and does not necessarily affect validation accuracy [9]. Systematic errors, on the other hand, will be incorporated into the model and propagate into the predictions from new spectra, leading to biased predictions and lower validation accuracies when spectral SOC estimates are compared with different SOC reference data. Accordingly, studies employing SOC reference data from the same set of measurements for calibration and validation will be largely unaffected. If reference data from soil archives or spectral libraries were used for model calibration, however, systematic differences in laboratory SOC analysis, e.g., different elemental analyzers, could inflate the estimated RMSEs of VNIR and MIR models during validation.

The maximum measurement uncertainty due to either the spectral input configuration (ΔRMSE < 0.4 g·kg^−1^) or reference SOC data (ΔRMSE < 0.3 g·kg^−1^) was, in any case, markedly smaller than the difference in predictive accuracy between the VNIR and MIR instruments (ΔRMSE ~1.4 g·kg^−1^). Predictive accuracies of the SOC models in our study were within expectations for laboratory soil spectroscopy applications, in general (e.g., reviews [39,43,44]), and recent studies employing portable VNIR and MIR instruments in particular (e.g., [4,5,14,41,42,45]). Greater predictive accuracies for MIR spectral data than VNIR spectra have also been widely documented [5,37,40,42,46] and can be attributed to the aforementioned presence of more specific and well-defined absorption features in the MIR. This was also evident from our PCA, which allowed a much more distinct separation of spectral samples by the soil parent material in the MIR domain. Despite the clear advantages of MIR spectroscopy for soil analysis, at least for data collected on finely ground samples, it is worth noting at this point that VNIR instruments are hardly redundant. Apart from the fact that portable VNIR instrumentation is still better adapted to field use on heterogeneous samples [5,40,41,42], recent studies have also emphasized the potential advantages of fusing VNIR and MIR spectral data for more accurate and robust SOC predictions [42,45,47].

The accuracy of the SOC models reported here, especially in MIR, would be sufficient for the routine application of spectroscopic methods in soil surveying [8]. Soil spectroscopy can, therefore, effectively complement standard analytical methods as soil spectra can be collected more quickly and thus at lower costs, with comparable training and experience requirements for the instrument operators.

In this context, we want to emphasize the complementary aspect of soil spectroscopy since an initial calibration of the spectra to the soil properties of interest within a specific region and for a particular set of soil types is required. The models from our study, for example, would be appropriate to estimate SOC contents of cropland soils, developed from the parent materials covered in the calibration, in western Rhineland-Palatinate. Predicting SOC beyond the support of the calibration sample, e.g., for grassland or forest soils with typically greater SOC contents, would, on the other hand, be ill-advised without re-calibration and validation.

Accordingly, VNIR and MIR spectroscopy are well-suited to collect large amounts of additional quantitative soil data more efficiently, with the aim to improve spatial and temporal coverage rather than to replace standard analytical methods entirely. Importantly, the efficiency gains that may be achieved in that regard scale well with the number of soil properties under study, as multiple soil parameters can be derived from the spectral data in addition to SOC. This includes, e.g., the soil clay mineral content or particle size distribution and, if applicable, CaCO_3_ contents, the determination of which with standard analytical methods is considerably more laborious and expensive than SOC by dry combustion. Unlike the laboratory analytical effort that steadily increases with each additional soil property under study, the cost and time required to collect and process the spectral data would remain essentially the same.

## 5. Conclusions

Our calibration-validation experiment with multiple spectral measurement series and analytical SOC datasets showed that spectral measurements collected in duplicate with portable VNIR and MIR instruments and their derived SOC predictions were highly reliable, comparable in reproducibility to the SOC measurements by dry combustion. Given the considerably more accurate SOC predictions and higher robustness against calibration sample selection of the MIR spectrometer, together with equally excellent reproducibility of spectral measurements with both instruments, the portable MIR instrument may be regarded as superior overall for soil analysis on fine-ground samples.

The uncertainty of SOC data associated with the dry combustion reference method also had little impact on SOC prediction with VNIR and MIR data and was limited to cases with biased reference data in the calibration and validation and vice versa. In the case of random reference errors, uncertainty in analytical reference measurements is thus likely absorbed in the spectral calibration for the most part. However, biased SOC estimates will propagate into the SOC predictions and give lower ‘apparent’ validation accuracies as a result. Accordingly, VNIR and MIR validation accuracies in studies where calibration and reference data are part of the same set of measurements are unlikely to be significantly affected by uncertainty in the reference method. Soil spectroscopy studies dealing with the estimation of SOC from models calibrated on archived soil data or spectral libraries, on the other hand, should take into account that biased spectral predictions might be partly the result of systematic differences in the reference data employed for model validation.

Collecting a larger number of VNIR or MIR spectra for SOC modeling, beyond the standard measurement in duplicate on a single prepared sample, further appears to have only limited potential in terms of increasing the accuracy of SOC predictions and may not be worth the additional workload required. Our results explicitly refer to fine-ground soil samples, which are likely to benefit portable MIR instruments more than VNIR spectrometers. The effects of repeated spectral measurements on SOC calibrations for less intensive soil pre-treatments, including on-site data collection, remain to be addressed in future studies.

In this context, in situ applications ‘on-the-go’ or in mobile laboratories represent the most significant potential advantage of portable VNIR and MIR devices. Investigating the reproducibility of VNIR and MIR measurements and models for data collected directly on the soil surface—in the field, or alternatively, in field moist or air-dried conditions—would thus mark a critical next step in advancing the use of portable spectrometers to efficiently collect large amounts of quantitative soil data.

## Figures and Tables

**Table 1:** Measurement and instrument parameters for collected VNIR and MIR series.

Set	Instrument	Co-Added Scans	Spectral Resolution	Sampling Interval
VNIR_1_	ASD FieldSpec 4	2 × 75	3 nm at 700 nm 30 nm at 1400/2100 nm	1.4 nm (350–1000 nm) 2 nm (1001–2500 nm)
VNIR_2_				
VNIR_3_				
MIR_1_	Agilent 4300	2 × 64	4 cm^−1^	1.86 cm^−1^ (4000–650 cm^−1^)
MIR_2_				
MIR_3_				

**Table 2:** Summary statistics of SOC measurements (g kg^−1^) from three different laboratories and their average (*n* = 75); Q_1_ = first Quartile, Q_3_ = third quartile, SD = standard deviation.

	Minimum	Q_1_	Median	Q_3_	Maximum	Mean	SD	Skewness
**Lab_1_**	6.16	11.16	14.50	23.03	35.06	17.01	7.73	0.52
**Lab_2_**	6.00	10.88	14.38	22.79	35.28	16.91	7.84	0.54
**Lab_3_**	6.37	11.37	14.88	24.27	36.26	17.60	8.06	0.52
**Lab_AVG_**	6.18	11.12	14.59	23.36	35.54	17.17	7.87	0.53

**Table 3:** Comparison of agreement between SOC measurements (g·kg^−1^) from three different laboratories and their average (*n* = 75); bias refers to the mean difference between row and column measurement series.

	RMSE				Bias				R^2^			
	Lab_1_	Lab_2_	Lab_3_	Lab_AVG_	Lab_1_	Lab_2_	Lab_3_	Lab_AVG_	Lab_1_	Lab_2_	Lab_3_	Lab_AVG_
**Lab_1_**	–	0.36	0.78	0.30	–	0.10	−0.59	−0.16	–	0.998	0.997	0.999
**Lab_2_**		–	0.80	0.32		–	−0.69	−0.26		–	0.998	0.999
**Lab_3_**			–	0.52			–	0.43			–	0.999

**Table 4:** Summary of spectral repeatability (*S_r_*) analysis for replicate VNIR and MIR measurement series 1–3; Q_1_ = first quartile, Q_3_ = third quartile, SD = standard deviation, CV_%_ = coefficient of variation.

	VNIR					MIR				
	Q_1_	Mean	Q_3_	SD	CV_%_	Q_1_	Mean	Q_3_	SD	CV_%_
***S_r_*** **(1)**	7499	13,523	17,485	8855	65.5	568	1350	1751	932	69.0
***S_r_*** **(2)**	9201	13,944	17,525	6907	49.5	983	1766	2404	895	50.7
***S_r_*** **(3)**	7533	14,797	19,355	8695	58.8	632	1165	1542	671	57.6
***S_r_*** **(1,2)**	18,023	32,187	39,730	22,100	68.7	1357	2729	3782	1775	65.0
***S_r_*** **(1,3)**	17,967	32,351	44,361	19,811	61.2	1363	2211	2941	1097	49.6
***S_r_*** **(2,3)**	14,440	27,615	39,002	17,805	64.5	1573	2308	2849	1116	48.4

**Table 5:** Validation accuracy of VNIR and MIR SOC models for the ‘best-case’ scenario with averaged spectral data from all measurement series (VNIR_AVG_ and MIR_AVG_) and averaged SOC reference data from all three laboratories. Statistics represent average values of 100 randomized runs of the nested cross-validation approach with two standard deviations of their respective distributions given in parentheses. RPD (ratio of performance to deviation) and RPIQ (ratio of performance to interquartile range) scores represent the ratios of reference SOC standard deviation and interquartile range, respectively, to the RMSE of the predicted SOC values.

	RMSE (g·kg^−1^)	R^2^	Bias (g·kg^−1^)	RPD	RPIQ
**VNIR**	2.57 (±0.50)	0.89 (±0.04)	0.12 (±0.39)	3.04 (±0.59)	4.67 (±0.91)
**MIR**	1.12 (±0.16)	0.98 (±0.01)	0.25 (±0.14)	7.01 (±0.99)	10.65 (±1.50)

**Table 6:** Validation accuracy (RMSE in g·kg^−1^) of VNIR and MIR SOC models for different combinations of calibration and validation (prediction) spectra using the laboratory-average SOC (Lab_AVG_) as reference data. Statistics represent average RMSE values of 100 randomized runs of the nested cross-validation approach with two standard deviations of their respective distributions given in parentheses.

Calibration Spectra *	Validation Spectra							
	VNIR_1_	VNIR_2_	VNIR_3_	VNIR_AVG_	MIR_1_	MIR_2_	MIR_3_	MIR_AVG_
**SPEC_1_**	2.91 (±0.44)	2.77 (±0.48)	2.83 (±0.44)	2.75 (±0.45)	1.36 (±0.19)	1.39 (±0.16)	1.34 (±0.13)	1.20 (±0.15)
**SPEC_2_**	2.73 (±0.49)	2.65 (±0.51)	2.71 (±0.47)	2.58 (±0.50)	1.43 (±0.21)	1.48 (±0.21)	1.30 (±0.21)	1.25 (±0.20)
**SPEC_3_**	2.91 (±0.45)	2.84 (±0.47)	2.86 (±0.49)	2.78 (±0.47)	1.47 (±0.18)	1.38 (±0.19)	1.45 (±0.17)	1.30 (±0.16)
**SPEC_AVG_**	2.77 (±0.49)	2.63 (±0.49)	2.70 (±0.50)	2.57 (±0.50)	1.37 (±0.19)	1.30 (±0.17)	1.31 (±0.16)	1.12 (±0.16)

* SPEC refers to VNIR and MIR, respectively.

**Table 7:** Validation accuracy (RMSE in g·kg^−1^) of VNIR and MIR SOC models for different combinations of calibration and validation laboratory SOC reference data using the averaged spectral data (VNIR_AVG_, MIR_AVG_) for model calibration and validation (prediction). Statistics represent average RMSE values of 100 randomized runs of the nested cross-validation approach with two standard deviations of their respective distributions given in parentheses.

**Calibration**	**Validation**							
		**VNIR_AVG_**				**MIR_AVG_**		
**SOC**	**Lab_1_**	**Lab_2_**	**Lab_3_**	**Lab_AVG_**	**Lab_1_**	**Lab_2_**	**Lab_3_**	**Lab_AVG_**
**Lab_1_**	2.56 (±0.50)	2.56 (±0.50)	2.68 (±0.47)	2.56 (±0.49)	1.13 (±0.16)	1.13 (±0.16)	1.37 (±0.16)	1.15 (±0.16)
**Lab_2_**	2.59 (±0.49)	2.59 (±0.49)	2.73 (±0.47)	2.60 (±0.48)	1.12 (±0.18)	1.12 (±0.18)	1.41 (±0.17)	1.16 (±0.17)
**Lab_3_**	2.71 (±0.53)	2.71 (±0.53)	2.65 (±0.51)	2.64 (±0.52)	1.37 (±0.19)	1.37 (±0.19)	1.24 (±0.17)	1.25 (±0.18)
**Lab_AVG_**	2.59 (±0.51)	2.59 (±0.51)	2.65 (±0.49)	2.57 (±0.50)	1.15 (±0.16)	1.15 (±0.16)	1.28 (±0.16)	1.12 (±0.16)

## Data Availability

The raw data are available on request to the authors.
